# A new improved generalized class of estimators for population distribution function using auxiliary variable under simple random sampling

**DOI:** 10.1038/s41598-023-30150-9

**Published:** 2023-04-03

**Authors:** Sohaib Ahmad, Kalim Ullah, Erum Zahid, Javid Shabbir, Muhammad Aamir, Huda M. Alshanbari, Abd Al-Aziz Hosni El-Bagoury

**Affiliations:** 1 Department of Statistics, Abdul Wali Khan University, Mardan, Pakistan; 2 Foundation University Medical College, Foundation University School of Health Sciences, DHA-I, Islamabad, 44000 Pakistan; 3 Department of Applied Mathematics and Statistics, Institute of Space Technalogy, Islamabad, Pakistan; 4 Department of Statistics, Quaid-I-Azam University, Islamabad, Pakistan; 5 Department of Statistics, University of Wah at Wah Cantt, Punjab, Pakistan; 6 Department of Mathematical Sciences, College of Science, Princess Nourah bint Abdulrahman University, P.O. Box 84428, Riyadh, 11671 Saudi Arabia; 7 Basic Sciences Department, Higher Institute of Engineering and Technology, EL-Mahala EL-Kobra, El-Mahalla El-Kubra, Egypt

**Keywords:** Engineering, Mathematics and computing, Applied mathematics, Computational science, Scientific data, Statistics

## Abstract

This article aims to suggest a new improved generalized class of estimators for finite population distribution function of the study and the auxiliary variables as well as mean of the usual auxiliary variable under simple random sampling. The numerical expressions for the bias and mean squared error (MSE) are derived up to first degree of approximation. From our generalized class of estimators, we obtained two improved estimators. The gain in second proposed estimator is more as compared to first estimator. Three real data sets and a simulation are accompanied to measure the performances of our generalized class of estimators. The MSE of our proposed estimators is minimum and consequently percentage relative efficiency is higher as compared to their existing counterparts. From the numerical outcomes it has been shown that the proposed estimators perform well as compared to all considered estimators in this study.

## Introduction

Generally it is a well-established notion that when the auxiliary variable is used appropriately in survey sampling, precision of the estimator is increased. Numbers of estimators exist in the literature for estimating different population parameters, such as mean, variance and total etc. but little attention has been paid to study the distribution (DF). The works on survey sampling discusses a diversity of procedures for incorporating the auxiliary variable via ratio, product, and regression methods of estimation. Numerous researchers have suggested various estimators by adequately adapting the auxiliary variable. These research findings can be investigated by Grover and Kaur^1^ suggested a generalized class of ratio type exponential estimators of population mean under linear transformation of auxiliary variable. Ahmad et al.^2^ discussed use of extreme values to estimate the finite population mean under PPS sampling scheme. Audu et al.^3^ suggested on the efficiency of almost unbiased mean imputation when population mean of auxiliary variable is unknown. Singh and Nigam^4^ discussed efficient method of estimating the finite population mean based on two auxiliary variables in the presence of non-response under stratified sampling. Shahzad et al.^5^ discussed estimation of the population mean by successive use of an auxiliary variable in median ranked set sampling. Singh et al.^6^ proposed some imputation methods to deal with the problems of missing data in two-occasion successive sampling. Aggarwal et al.^7^ discussed estimation of the population mean by developing a new estimator. Singh et al.^6^ suggested an exponential approach for estimating population mean using two auxiliary variables in stratified random sampling. Yadav et al.^8^ proposed new modified ratio type estimator of the population mean using the known median of the study variable. Pal and Singh^9^ discussed about estimation of finite population mean using auxiliary information in presence of non-response. Pal and Singh^10^ proposed an efficient new approach for estimating the general parameter using auxiliary variable in sample surveys. Shahzad et al.^11^ suggested quantile regression-ratio-type estimators for mean estimation under complete and partial auxiliary information. Zaman et al.^12^ discussed robust ratio‐type estimators for finite population mean in simple random sampling. Singh et al.^13^ discussed some efficient classes of estimators of population mean in two-phase successive sampling under random non response. Singh and Khalid^14^ proposed some imputation methods to compensate with non-response for estimation of population mean in two-occasion successive sampling. Irfan et al.^15^ proposed difference-type-exponential estimators based on dual auxiliary information under simple random sampling. Riyaz et al.^16^ discussed generalized exponential ratio estimator of population mean using two auxiliary variables in simple random sampling with an application to agricultural data. Zaman et al.^17^ proposed robust regression-ratio-type estimators of the mean utilizing two auxiliary variables. For assessing the finite population mean, researchers have suggested various improved ratio, product, and regression type estimators in their work.

There are numerous estimators available in literature for estimating different finite population parameters under various sampling designs, but the study based on distribution function (DF) has received less attention. When it needs to determine the percentage of particular values that are small or equal to the threshold value, the estimation of a finite population DF becomes necessary. For example a doctor is interested to distinguish the percentage of the population who consume at least 30% of their energy from a dietary cholesterol. A soil researcher is interested to determine how many peoples in a developing nation live below the poverty level. In all these circumstances, survey sampling significantly relies on estimation of finite population distribution function. In the area of DF, some significant work includes Chambers and Dunstan^18^ suggested estimating distribution functions from survey data. Chambers et al.^19^ discussed properties of estimators of the finite population distribution function. Dorfman^20^ discussed a comparison of design‐based and model‐based estimators of the finite population distribution function. Ahmad and Abu-Dayyah^21^ suggested estimation of finite-population distribution function using multivariate auxiliary information. Singh et al.^22^ discussed a family of estimators of finite-population distribution function using auxiliary information. Onsongo et al.^23^ discussed bias reduction technique for estimating finite population distribution function under simple random sampling without replacement. Ahmad et al.^24^ discussed a new generalized class of exponential factor-type estimators for population distribution function using two auxiliary variables.

The continuing of the article is ordered as follows. Notations and symbols of the article are given in “Notations and symbols” section. The literature evaluation of the various estimators based on simple random sampling is introduced in “Review of existing estimators” section. The suggested a generalized class of estimators is given in Section 4. In Section 5, numerical investigation and data description are provided. In Section 6, a simulation study is given. Section 7 provides discussion of the article. Finally conclusion of the article is presented in Section 8.

## Notations and symbols

Let a finite population ℧ = (${{\mho }}_{1},{{\mho }}_{2},\dots ,{{\mho }}_{N}$) consist of *N* identified and distinct units. A sample of size *n* is selected from ℧ by using simple random sampling without replacement. Let ${Y}_{i}$ and ${X}_{i}$ be the values of the study variable (*Y*) and the auxiliary variable (*X*) respectively. Let $\overline{Y }$ and $\overline{X }$ be the population means respectively corresponding to the sample means $\overline{y }$ and $\overline{x }$. The indicator function of the study variable and the auxiliary variable are represented by $I({Y}_{i}\le \mathrm{Y})$, and $I({X}_{i}\le \mathrm{X})$. Let the finite population distribution function for the population and sample of the study variable and the auxiliary variable are: $\mathtt{F}(y)$=$\frac{\sum_{i=1}^{N}I({Y}_{i}\le \mathrm{Y})}{N}$ , $\widehat{\mathtt{F}}$(y) =$\frac{\sum_{i=1}^{n}I({Y}_{i}\le \mathrm{Y})}{n}$ , $\mathtt{F}(x)$=$\frac{\sum_{i=1}^{N}I({X}_{i}\le \mathrm{X})}{N}$ , $\widehat{\mathtt{F}}$(x) = $\frac{\sum_{i=1}^{n}I({X}_{i}\le \mathrm{X})}{n}$.

To attain the bias and mean square error we consider the following relative error terms:

$$\xi_{0} = \frac{{\hat{\user2{F}}_{y} - {\varvec{F}}_{y} }}{{{\varvec{F}}_{y} }},\xi_{1} = \frac{{\hat{\user2{F}}_{x} - {\varvec{F}}_{x} }}{{{\varvec{F}}_{x} }},\xi_{2} = \frac{{\overline{x} - \overline{X}}}{{\overline{X}}},{\text{such}}\,{\text{that}}$$

$${\text{E}}(\xi_{0}^{2} ) = \, \lambda C_{{{\varvec{F}}\left( y \right){ }}}^{2} = \Theta_{200} ,{\text{E }}(\xi_{1}^{2} ) = \lambda C_{{{\varvec{F}}\left( x \right){ }}}^{2} = \Theta_{020} ,{\text{E }}(\xi_{2}^{2} ) = \lambda C_{{x{ }}}^{2} = \Theta_{002} ,$$

$${\text{E }}(\xi_{0} \xi_{1} ) = \lambda \rho_{{{\varvec{F}}\left( y \right){\varvec{F}}\left( x \right)}} C_{{{\varvec{F}}\left( y \right)}} C_{{{\varvec{F}}\left( x \right)}} = \Theta_{110} ,{\text{E}}(\xi_{0} \xi_{2} ) = \lambda \rho_{{{\varvec{F}}\left( y \right)x}} C_{{{\varvec{F}}\left( y \right)}} C_{x} = \Theta_{101} ,$$

$${\text{E }}(\xi_{1} \xi_{2} ) = \lambda \rho_{{{\varvec{F}}\left( x \right)x}} C_{{{\varvec{F}}\left( x \right)}} C_{x} = \Theta_{011}$$

$$S_{{{\varvec{F}}\left( y \right){ }}}^{2} = \frac{{\mathop \sum \nolimits_{i = 1}^{N} \left\{ {I\left( {Y_{i} \le {\text{Y}}} \right) - {\varvec{F}}\left( y \right)} \right\}^{2} }}{N - 1},S_{{{\varvec{F}}\left( x \right){ }}}^{2} = \frac{{\mathop \sum \nolimits_{i = 1}^{N} \left\{ {I\left( {X_{i} \le {\text{X}}} \right) - {\varvec{F}}\left( x \right)} \right\}^{2} }}{N - 1},S_{x}^{2} = \frac{{\mathop \sum \nolimits_{i = 1}^{N} \left\{ {X_{i} - \overline{X}} \right\}^{2} }}{N - 1}$$

$$C_{{{\varvec{F}}\left( y \right){ }}}^{2} = \frac{{S_{{{\varvec{F}}\left( y \right){ }}}^{2} }}{{{\varvec{F}}^{2} \left( y \right)}}, C_{{{\varvec{F}}\left( x \right){ }}}^{2} = \frac{{S_{{{\varvec{F}}\left( x \right){ }}}^{2} }}{{{\varvec{F}}^{2} \left( x \right)}}, C_{{x{ }}}^{2} = \frac{{S_{{x{ }}}^{2} }}{{\overline{X}^{2} }}$$

$$\rho_{{{\varvec{F}}\left( y \right){\varvec{F}}\left( x \right)}} = \mathop \sum \limits_{i = 1}^{N} \left[ {\left\{ {I\left( {Y_{i} \le {\text{Y}}} \right) - {\varvec{F}}\left( y \right)} \right\}\left\{ {I\left( {X_{i} \le {\text{X}}} \right) - {\varvec{F}}\left( x \right)} \right\}} \right],$$

$$\rho_{{{\varvec{F}}\left( y \right)x}} = \mathop \sum \limits_{i = 1}^{N} \left[ {\left\{ {I\left( {Y_{i} \le {\text{Y}}} \right) - {\varvec{F}}\left( y \right)} \right\}\left\{ {X_{i} - \overline{X}} \right\} } \right],$$

$$\rho_{{{\varvec{F}}\left( x \right)x}} = \mathop \sum \limits_{i = 1}^{N} \left[ {\left\{ {I\left( {X_{i} \le {\text{X}}} \right) - {\varvec{F}}\left( x \right)} \right\}\left\{ {X_{i} - \overline{X}} \right\}} \right],$$

$$\lambda = \left( {\frac{1}{n} - \frac{1}{N}} \right).$$

## Review of existing estimators

In this section, we present some exisitng estimators of finite population DF.

1. The ususal estimator of $\mathtt{F}(y)$, is given by:

1 $${\widehat{\mathtt{F}}}_{usual} = \frac{1}{n}\sum_{i=1}^{n}I\left({Y}_{i}\le \mathrm{Y}\right).$$

The variance of ${\widehat{\mathtt{F}}}_{usual}$, is given by:

2 $$\mathrm{Var}\left({\widehat{\mathtt{F}}}_{usual}\right) ={F}_{(y)}^{2}{\Theta }_{200}.$$

2. Cochran^25^ presented the following estimator:

3 $$\widehat{\mathtt{F}}\left(\mathrm{R}\right)= \widehat{\mathtt{F}}\left(y\right)\left(\frac{\mathtt{F}\left(x\right)}{\widehat{\mathtt{F}}\left(x\right)}\right).$$

The bias and MSE of $\widehat{\mathtt{F}}(\mathrm{R})$, are given by

$$\mathrm{Bias}\left(\widehat{\mathtt{F}}(\mathrm{R}) \right)\cong \mathtt{F}(y)\left({\Theta }_{020}-{\Theta }_{110}\right),$$

4 $$\mathrm{MSE}\left(\widehat{\mathtt{F}}(\mathrm{R}) \right)\cong {\mathtt{F}}^{2}\left(y\right)\left({\Theta }_{200}+{\Theta }_{020}-2{\Theta }_{110}\right).$$

3. Murthy^26^ suggested the following estimator of $\mathtt{F}(y)$ as:

5 $$\widehat{\mathtt{F}}\left(\mathrm{P}\right)= \widehat{\mathtt{F}}\left(y\right)\left(\frac{\widehat{\mathtt{F}}\left(x\right)}{\mathtt{F}\left(x\right)}\right).$$

The bias and MSE of $\widehat{\mathtt{F}}\left(\mathrm{P}\right),$ are given by

$$\mathrm{Bias}\left(\widehat{\mathtt{F}}(\mathrm{P}) \right)\cong \mathtt{F}(y){\Theta }_{110},$$

6 $$\mathrm{MSE}\left(\widehat{\mathtt{F}}(\mathrm{P}) \right)\cong {\mathtt{F}}^{2}\left(y\right)\left({\Theta }_{200}+{\Theta }_{020}+2{\Theta }_{110}\right).$$

4. Haq and Shabbir^27^ suggested the following two estimators, which are given by:

7 $${\widehat{\mathtt{F}}}_{HS,1}=\left[{\psi}_{1} {\widehat{\mathtt{F}}}_{BT,A}+{\psi}_{2}\left\{\mathtt{F}\left(x\right)-\widehat{\mathtt{F}}(x)\right\}\right]\mathrm{ exp}\left(\frac{\mathtt{F}(x)-\widehat{\mathtt{F}}(x)}{\widehat{\mathtt{F}}(x)+\mathtt{F}(x)}\right)$$

where ${\psi}_{\mathrm{i}}$ (*i* = 1,2,3,4) are constants and

$$\hat{\user2{F}}_{BT,A} = \frac{{\hat{\user2{F}}\left( y \right)}}{2}\left[ {{\text{exp}}\left( {\frac{{{\varvec{F}}\left( x \right) - \hat{\user2{F}}\left( x \right)}}{{\hat{\user2{F}}\left( x \right) + {\varvec{F}}\left( x \right)}}} \right) + {\text{exp}}\left( {\frac{{\hat{\user2{F}}\left( x \right) - {\varvec{F}}\left( x \right)}}{{\hat{\user2{F}}\left( x \right) + {\varvec{F}}\left( x \right)}}} \right)} \right].$$

The bias of ${\widehat{\mathtt{F}}}_{HS,1}$ is given by:

$${\text{Bias}}(\hat{\user2{F}}_{HS,1} ) \cong { }\frac{1}{2}\left[ { - 2 {\varvec{F}}\left( y \right) + {\varvec{F}}\left( y \right)\left\{ {2 + \lambda C_{{{\varvec{F}}\left( x \right)}} \left( {2C_{{{\varvec{F}}\left( x \right)}} - \rho_{{{\varvec{F}}\left( y \right){\varvec{F}}\left( x \right)}} } \right)} \right\}\psi_{1} + {\varvec{F}}\left( x \right)\lambda C_{{{\varvec{F}}\left( x \right)}}^{2} \psi_{2} } \right]$$

where ${\psi}_{1}$ and ${\psi}_{2}$ are constants. The optimum value of ${\psi}_{1}$ and ${\psi}_{2}$ are given by:

$${\psi}_{1(\mathrm{opt})}=\frac{4}{\left[4+\lambda {C}_{\mathtt{F}\left(x\right)}^{2}-4\lambda {C}_{\mathtt{F}\left(y\right)}^{2}(-1+{\rho }_{\mathtt{F}\left(y\right)\mathtt{F}\left(x\right)}^{2})\right]},$$

$${\psi}_{2(\mathrm{opt})}=\frac{\mathtt{F}\left(y\right)}{2\mathtt{F}\left(x\right)}\left[1+\left(\frac{-8{C}_{\mathtt{F}\left(x\right)}+8{C}_{\mathtt{F}\left(y\right){\rho }_{\mathtt{F}\left(y\right)\mathtt{F}\left(x\right)}}}{{C}_{\mathtt{F}\left(x\right)}\left\{4+\lambda {C}_{\mathtt{F}\left(x\right)}^{2}-4\lambda {C}_{\mathtt{F}\left(y\right)}^{2}\left(-1+{\rho }_{\mathtt{F}\left(y\right)\mathtt{F}\left(x\right)}^{2}\right)\right\}}\right)\right]$$

The minimum MSE of ${\widehat{\mathtt{F}}}_{HS,1}$ is given by:

8 $${\text{MSE}}\left( {\hat{\user2{F}}_{HS,1} } \right) _{min} \cong \frac{{\lambda {\varvec{F}}^{2} \left( y \right)\left[ { - \lambda C_{{{\varvec{F}}\left( x \right)}}^{4} + 4\left( {1 - \rho_{{{\varvec{F}}\left( y \right){\varvec{F}}\left( x \right)}}^{2} } \right)\left( { - 4 + \lambda C_{{{\varvec{F}}\left( x \right)}}^{4} } \right){\varvec{F}}^{2} \left( y \right)} \right]}}{{4\left[ {4 + \lambda C_{{{\varvec{F}}\left( x \right)}}^{4} - 4\lambda C_{{{\varvec{F}}\left( y \right)}}^{4} \left( { - 1 - \rho_{{{\varvec{F}}\left( y \right){\varvec{F}}\left( x \right)}}^{2} } \right)} \right]}}$$

and

9 $${\widehat{\mathtt{F}}}_{HS,2}=\frac{{\psi}_{3}}{2}{\widehat{\mathtt{F}}}_{BT,A}\left\{\frac{\mathtt{F}(x)}{\widehat{\mathtt{F}}(x)}+\frac{\widehat{\mathtt{F}}(x)}{\mathtt{F}(x)}\right\}+{\psi}_{4}\left\{\mathtt{F}\left(x\right)-\widehat{\mathtt{F}}(x)\right\}\mathrm{exp}\left(\frac{\mathtt{F}(x)-\widehat{\mathtt{F}}(x)}{\widehat{\mathtt{F}}(x)+\mathtt{F}(x)}\right)$$

The bias of ${\widehat{\mathtt{F}}}_{HS,2}$ are given by:

$${\text{Bias}}(\hat{\user2{F}}_{HS,2} ) \cong { }\frac{1}{2}\left[ { - 2 {\varvec{F}}\left( y \right) + {\varvec{F}}\left( y \right)\left\{ {2 + \lambda C_{{{\varvec{F}}\left( x \right)}} \left( {2C_{{{\varvec{F}}\left( x \right)}} - \rho_{{{\varvec{F}}\left( y \right){\varvec{F}}\left( x \right)}} C_{{{\varvec{F}}\left( y \right)}} } \right)} \right\}\psi_{3} + {\varvec{F}}\left( x \right)\lambda C_{{{\varvec{F}}\left( x \right)}}^{2} \psi_{4} } \right]$$

where ${\psi}_{3}$ and ${\psi}_{4}$ are constants. The optimum value of ${\psi}_{3}$ and ${\psi}_{4}$ are given by:

$${\psi}_{3(\mathrm{opt})}=\frac{4+2\lambda {C}_{\mathtt{F}\left(y\right)}^{2}}{\left[4+5\lambda {C}_{\mathtt{F}\left(x\right)}^{2}-4\lambda {C}_{\mathtt{F}\left(y\right)}^{2}(-1+{\rho }_{\mathtt{F}\left(y\right)\mathtt{F}\left(x\right)}^{2})\right]},$$

$${\psi}_{4(\mathrm{opt})}=\left[\frac{\mathtt{F}\left(y\right)\left\{8{{C}_{\mathtt{F}\left(y\right)}\rho }_{\mathtt{F}\left(y\right)\mathtt{F}\left(x\right)}+{C}_{\mathtt{F}\left(x\right)}\left\{-4+\lambda \left({C}_{\mathtt{F}\left(x\right)}^{2}+4{\rho }_{\mathtt{F}\left(y\right)\mathtt{F}\left(x\right)}-{C}_{\mathtt{F}\left(y\right)}{C}_{\mathtt{F}\left(x\right)}\left(-1+{\rho }_{\mathtt{F}\left(y\right)\mathtt{F}\left(x\right)}^{2}\right)\right)\right\}\right\}}{2\mathtt{F}\left(x\right){C}_{\mathtt{F}\left(x\right)}\left[4+5\lambda {C}_{\mathtt{F}\left(x\right)}^{2}-4\lambda {C}_{\mathtt{F}\left(y\right)}^{2}\left(-1+{\rho }_{\mathtt{F}\left(y\right)\mathtt{F}\left(x\right)}^{2}\right)\right]}\right]$$

The minimum MSE of ${\widehat{\mathtt{F}}}_{HS,1}$ at the optimum values of ${\psi}_{3}$ and ${\psi}_{4}$, is given by:

10 $${\text{MSE}}\left( {\hat{\user2{F}}_{HS,2} } \right) _{min} \cong \frac{{\lambda {\varvec{F}}^{2} \left( y \right)\left[ { - 9\lambda C_{{{\varvec{F}}\left( x \right)}}^{4} + 4\left( {1 + \rho_{{{\varvec{F}}\left( y \right){\varvec{F}}\left( x \right)}}^{2} } \right)\left( { - 4 + \lambda C_{{{\varvec{F}}\left( x \right)}}^{2} } \right){\varvec{F}}^{2} \left( y \right)} \right]}}{{4\left[ {4 + 5\lambda C_{{{\varvec{F}}\left( x \right)}}^{4} - 4\lambda C_{{{\varvec{F}}\left( y \right)}}^{4} \left( { - 1 - \rho_{{{\varvec{F}}\left( y \right){\varvec{F}}\left( x \right)}}^{2} } \right)} \right]}}$$

5. The regression estimator of $\mathtt{F}\left(y\right)$, is given by:

11 $${\widehat{\mathtt{F}}}_{Reg}= \widehat{\mathtt{F}}\left(y\right)+{\psi}_{5}\left[\mathtt{F}\left(x\right)-\widehat{\mathtt{F}}\left(x\right)\right],$$

where ${\psi}_{5}$ is constant. The optimum value of ${\psi}_{5}$ is

12 $${\psi}_{5(\mathrm{opt})} =\frac{\mathtt{F}\left(y\right){\Theta }_{110}}{\mathtt{F}\left(y\right){\Theta }_{020}}$$

The minimum variance of ${\widehat{\mathtt{F}}}_{Reg}$, is given by:

13 $${\mathrm{Var}\left({\widehat{\mathtt{F}}}_{Reg}\right)}_{min} = {\mathtt{F}}^{2}\left(y\right){\Theta }_{200}\left(1-{\rho }_{\mathtt{F}\left(y\right)\mathtt{F}\left(x\right)}^{2}\right).$$

where $${\rho }_{\mathtt{F}\left(y\right)\mathtt{F}\left(x\right)}$$=$$\frac{{\Theta }_{110}}{\sqrt{{\Theta }_{110}}\sqrt{{\Theta }_{020}}}$$

6. Following Bahl and Tuteja ^28^, exponential estimators of $\mathtt{F}(y)$, are given by:

14 $${\widehat{\mathtt{F}}}_{BTR}=\widehat{\mathtt{F}}(y)\mathrm{exp}\left(\frac{\mathtt{F}(x)-\widehat{\mathtt{F}}(x)}{\widehat{\mathtt{F}}(x)+\mathtt{F}(x)}\right)$$

and

15 $${\widehat{\mathtt{F}}}_{BTP}=\widehat{\mathtt{F}}\left(y\right)\mathrm{exp}\left(\frac{\widehat{\mathtt{F}}\left(x\right)-\mathtt{F}\left(x\right)}{\widehat{\mathtt{F}}\left(x\right)+\mathtt{F}\left(x\right)}\right).$$

The bias and MSE of ${\widehat{\mathtt{F}}}_{BTR}$ and ${\widehat{\mathtt{F}}}_{BTP},$ are given by

$$\mathrm{Bias}({\widehat{\mathtt{F}}}_{BTR})\cong \mathtt{F}\left(y\right)\left(\frac{3}{8}{\Theta }_{020}-\frac{1}{2}{\Theta }_{110}\right),$$

16 $$\mathrm{MSE}({\widehat{\mathtt{F}}}_{BTR})\cong \frac{{\mathtt{F}}^{2}(y)}{4}\left(4{\Theta }_{200}+{\Theta }_{020}-4{\Theta }_{110}\right)$$

and

$$\mathrm{Bias}({\widehat{\mathtt{F}}}_{BTP})\cong \mathtt{F}\left(y\right)\left(\frac{1}{2}{\Theta }_{110}-\frac{1}{8}{\Theta }_{020}\right),$$

17 $$\mathrm{MSE}\left({\widehat{\mathtt{F}}}_{BTP}\right)\cong \frac{{\mathtt{F}}^{2}\left(y\right)}{4}\left(4{\Theta }_{200}+{\Theta }_{020}+4{\Theta }_{110}\right).$$

7. Shabbir and Gupta ^29^ suggested estimator for $\mathtt{F}$(y):

18 $${\widehat{\mathtt{F}}}_{SG} = {\psi}_{6}\widehat{\mathtt{F}}\left(y\right)+{\psi}_{7}\left[\mathtt{F}\left(x\right)-\widehat{\mathtt{F}}\left(x\right)\right],$$

where ${\psi}_{6}$ and ${\psi}_{7}$ are unknown constants.

The optimum values are

$${\psi}_{6}=\frac{{\Theta }_{020}}{8}\left[\frac{8-{\Theta }_{020}}{{\Theta }_{020}+{\Theta }_{200}{\Theta }_{020}-{\Theta }_{220}^{2}}\right],$$

and

$${\psi}_{7}=\frac{\widehat{\mathtt{F}}(y)}{8\mathtt{F}\left(x\right)}\left[\frac{-4{\Theta }_{200}+{\Theta }_{020}+8{\Theta }_{220}-{\Theta }_{220}{\Theta }_{020+}4{\Theta }_{200}-4{\Theta }_{220}^{2}}{{\Theta }_{200}+{\Theta }_{200}{\Theta }_{020-}{\Theta }_{220}^{2}}\right].$$

The bias and minimum MSE of ${\widehat{\mathtt{F}}}_{SG}$, are given as:

$$\mathrm{Bias}\left({\widehat{\mathtt{F}}}_{SG}\right)\cong \mathtt{F}\left(y\right)\left({\psi}_{6}-1\right)$$

19 $${\mathrm{MSE}\left({\widehat{\mathtt{F}}}_{SG}\right)}_{min}\cong \frac{{\mathtt{F}}^{2}(y)}{64}\left[\frac{-4{\Theta }_{020}^{2}-16{\Theta }_{020}(1-{\rho }_{\mathtt{F}\left(y\right)\mathtt{F}\left(x\right)}^{2})({\Theta }_{020}-4)}{1+{\Theta }_{200}(1-{\rho }_{\mathtt{F}\left(y\right)\mathtt{F}\left(x\right)}^{2})}\right].$$

8. Swain^30^ suggested th e following estimator for $\mathtt{F}$(y) and is, given by:20

where $\mathrm{\alpha }$ is the unknown constant; and , $Q$ and $\sigma$ are scaler quantity.

The bias of ${\widehat{\mathtt{F}}}_{SW}$ is given by:

$${\text{Bias}}(\hat{\user2{F}}_{SW} ) = \user2{ F}\left( y \right)\left[ {A_{1} \Theta_{110} + A_{2} \Theta_{020} } \right],$$

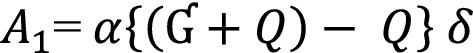



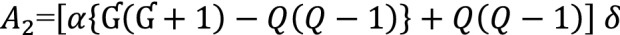


The optimum value of $\alpha$ i.e. ${\alpha }_{opt}$ is given by:
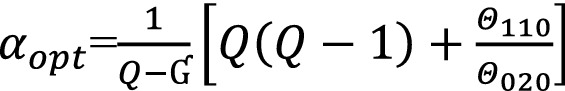


The minimum MSE of ${\widehat{\mathtt{F}}}_{SW}$ is given by

21 $$\mathrm{MSE}\left({\widehat{\mathtt{F}}}_{SW}\right)\cong {\mathtt{F}}^{2}\left(y\right){\Theta }_{200}\left(1-{\rho }_{\mathtt{F}\left(y\right)\mathtt{F}\left(x\right)}^{2}\right)={\mathrm{Var}\left({\widehat{\mathtt{F}}}_{Reg}\right)}_{min}$$

9. Chami et al. ^31^ suggested the following estimator is given by:

22 $$\hat{\user2{F}}_{C} = \hat{\user2{F}}\left( y \right)\left\{ {\psi_{9} \frac{{\left( {1 - \beta_{1} } \right)\hat{\user2{F}}\left( x \right) + \beta_{1} {\varvec{F}}\left( x \right)}}{{\beta_{1} \hat{\user2{F}}\left( x \right) + \left( {1 - \beta_{1} } \right){\varvec{F}}\left( x \right)}}} \right\} + \, ({1} - \psi_{9} )\left\{ {\frac{{\beta_{1} \hat{\user2{F}}\left( x \right) + \left( {1 - \beta_{1} } \right){\varvec{F}}\left( x \right)}}{{{\upalpha }\left( {1 - \beta_{1} } \right) + \beta_{1} {\varvec{F}}\left( x \right)}}} \right\}$$

where ${\psi}_{9}$ and ${\beta }_{1}$ are constants.

The bias of ${\widehat{\mathtt{F}}}_{C}$ is given by:

$${\text{Bias}}\left( {\hat{\user2{F}}_{C} { }} \right) = {\varvec{F}}\left( y \right)\left[ {\left( {1 - 2\beta_{1} } \right)\left( {2\psi_{9} - 1} \right)\Theta_{110} + \left( {1 - 2\beta_{1} } \right)\left( {1 - \beta_{1} - \psi_{9} } \right)\Theta_{020} } \right].$$

Putting the values of ${\psi}_{9}$ and ${\beta }_{1}$ we get the minimum variance of ${\widehat{\mathtt{F}}}_{C}$, and is equal to ${\mathrm{Var}\left({\widehat{\mathtt{F}}}_{Reg}\right)}_{min}$.

10. Yadav et al.^32^ suggested the following estimator is given by:

23 $${\widehat{\mathtt{F}}}_{YG}=\left[{\mathrm{\psi}}_{11}\widehat{\mathtt{F}}\left(y\right)+{\mathrm{\psi}}_{12}\left\{\mathtt{F}\left(x\right)-\widehat{\mathtt{F}}\left(x\right)\right\}\right]\left[\gamma \left(\frac{\alpha \mathtt{F}\left(x\right)+\beta }{\alpha \widehat{\mathtt{F}}\left(x\right)+\beta }\right)+(1-\gamma )\left(\frac{\alpha (\mathtt{F}\left(x\right)-\widehat{\mathtt{F}}\left(x\right))}{\alpha \left(\mathtt{F}\left(x\right)-\widehat{\mathtt{F}}\left(x\right)\right)+\beta }\right)\right].$$

where ${\psi}_{11}$ and ${\psi}_{12}$ are constants, and $\gamma$ is the scaler quantity.

The optimum values are:
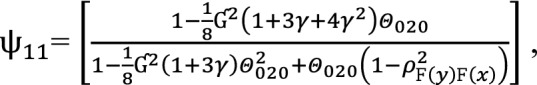


and
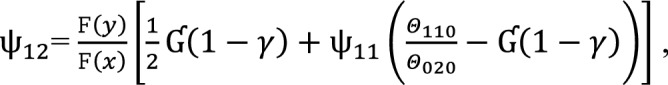



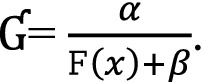


The bias of ${\widehat{\mathtt{F}}}_{YG}$ is given by
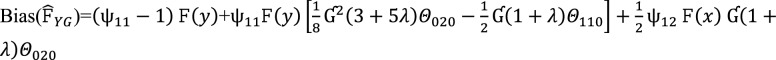


The minimum MSE of ${\widehat{\mathtt{F}}}_{YG}$ is

24 $${\mathrm{MSE}({\widehat{\mathtt{F}}}_{YG})}_{min}\cong {\mathtt{F}}^{2}(y)\left[\frac{\frac{1}{{\mathtt{F}}^{2}\left(y\right)}{\mathrm{Var}}_{\mathrm{min}}\left({\widehat{\mathtt{F}}}_{Reg}\right)(1-{\Theta }_{020})}{1-{\Theta }_{020}+\frac{1}{{\mathtt{F}}^{2}\left(y\right)}{\mathrm{Var}}_{\mathrm{min}}\left({\widehat{\mathtt{F}}}_{Reg}\right)}\right]$$

11. Yaqub and Shabbir^33^ suggetsed the following estimator, given by:

25 $$\begin{aligned}{\widehat{\mathtt{F}}}_{YS} & = \mathtt{F}\left(y\right)\left[\left\{{\psi}_{14 \widehat{\mathtt{F}}\left(y\right)+}{\psi}_{15}\left(\mathtt{F}\left(x\right)-\widehat{\mathtt{F}}\left(x\right)\right)\right\}\left(\frac{\alpha \mathtt{F}\left(x\right)+\beta }{\alpha \widehat{\mathtt{F}}\left(x\right)+2\beta }\right)\right]\\ & \quad \left[\frac{1}{2}\mathrm{exp}\left(\frac{\alpha (\mathtt{F}\left(x\right)-\widehat{\mathtt{F}}\left(x\right))}{\alpha \left(\mathtt{F}\left(x\right)-\widehat{\mathtt{F}}\left(x\right)\right)+2\beta }\right)+\frac{1}{2}\mathrm{exp}\left(\frac{\alpha (\widehat{\mathtt{F}}\left(x\right)-\mathtt{F}\left(x\right))}{\alpha \left(\widehat{\mathtt{F}}\left(x\right)-\mathtt{F}\left(x\right)\right)+\beta }\right)\right]\end{aligned}$$

where ${\psi}_{14}$ and ${\psi}_{15}$ are constants.

The bias of ${\widehat{\mathtt{F}}}_{YS}$ is given by:

$${\text{Bias}} = \left( {\psi_{14} - 1} \right)\user2{ F}\left( y \right)\left( {\hat{\user2{F}}_{YS} } \right) + \frac{9}{8}\psi_{14} \user2{ F}\left( y \right)\theta^{2} \Theta_{020} - \psi_{14} \user2{ F}\left( y \right)\theta \Theta_{110} + \psi_{15} \user2{ F}\left( x \right)\theta \Theta_{020}$$

The optimum values are ${\psi}_{14}$ and ${\psi}_{15}$ are given by:

$${\psi}_{14(\mathrm{opt})}=\frac{{\Theta }_{020}}{2}\left[\frac{1+7(1-{\Theta }_{020})}{{\Theta }_{020}^{2}+4\left\{{\Theta }_{020}(1-{\rho }_{\mathtt{F}\left(y\right)\mathtt{F}\left(x\right)}^{2})\right\}-{\Theta }_{110}^{2}}\right]$$

$${\psi}_{15(\mathrm{opt})}\frac{\mathtt{F}\left(y\right)}{\mathtt{F}\left(x\right)}\left[\frac{{\Theta }_{110 +\left(7{\Theta }_{110-}8 {\Theta }_{020}\right)\left(1-{\Theta }_{020}\right)+ 8 {\Theta }_{200}{\Theta }_{020}-8{\Theta }_{110}^{2}}}{{\Theta }_{020}^{2}+4{\Theta }_{020}(1-{\Theta }_{020}+{\Theta }_{200})-{\Theta }_{110}^{2}}\right]$$

The minimum MSE of ${\widehat{\mathtt{F}}}_{YS}$ is given by:

26 $$\mathrm{MSE}({\widehat{\mathtt{F}}}_{YS})\cong \frac{{\mathtt{F}}^{2}(y)}{16}\left[\frac{\frac{64}{{\mathtt{F}}^{2}\left(y\right)} \left(1-{\Theta }_{020}\right)\mathrm{MSE}\left({\widehat{\mathtt{F}}}_{Reg}\right)-{\Theta }_{020}^{2}}{{\Theta }_{020}+4\left(1-{\Theta }_{200}\right)+2{\Theta }_{110}+\frac{4}{{\mathtt{F}}^{2}\left(y\right)}\mathrm{MSE}\left({\widehat{\mathtt{F}}}_{Reg}\right)}\right]$$

12. Muneer et al.^34^ suggested the following estimator, given by:

27 $${\widehat{\mathtt{F}}}_{SM}=\widehat{\mathtt{F}}\left(y\right)\left[{\psi}_{16}\left(\frac{\mathtt{F}\left(x\right)}{\widehat{\mathtt{F}}\left(x\right)}\right)+{\psi}_{17}\left(\frac{\widehat{\mathtt{F}}\left(x\right)}{\mathtt{F}\left(x\right)}\right)\right]\mathrm{exp}\left(\frac{(\mathtt{F}\left(x\right)-\widehat{\mathtt{F}}\left(x\right))}{\left(\mathtt{F}\left(x\right)+\widehat{\mathtt{F}}\left(x\right)\right)}\right),$$

where ${\psi}_{16}$ and ${\psi}_{17}$ are constants. The optimum values are:

$${\psi}_{16(\mathrm{opt})}=\frac{1}{8}\left[\frac{16{\Theta }_{110}^{2}+6{\Theta }_{110}{\Theta }_{020}-24{\Theta }_{110}{\Theta }_{020}-16{\Theta }_{200}{\Theta }_{020}-{\Theta }_{020}^{2}-16{\Theta }_{110}-8{\Theta }_{020}}{16{\Theta }_{110}^{2}-16{\Theta }_{110}{\Theta }_{020}-4{\Theta }_{200}{\Theta }_{020}+{\Theta }_{020}^{2}-4{\Theta }_{020}}\right],$$

$${\psi}_{17(\mathrm{opt})}=\frac{1}{8}\left[\frac{48{\Theta }_{110}^{2}+16{\Theta }_{110}{\Theta }_{020}-72{\Theta }_{110}{\Theta }_{020}+16{\Theta }_{200}{\Theta }_{020}+21{\Theta }_{020}^{2}+16{\Theta }_{110}-24{\Theta }_{020}}{16{\Theta }_{110}^{2}-16{\Theta }_{110}{\Theta }_{020}-4{\Theta }_{200}{\Theta }_{020}+{\Theta }_{020}^{2}-4{\Theta }_{020}}\right]$$

The bias of ${\widehat{\mathtt{F}}}_{SM}$ is given by:

$$\mathrm{Bias}\left({\widehat{\mathtt{F}}}_{SM}\right)=\mathtt{ }\mathtt{F}\left(y\right)\left[\left({\psi}_{16}+{\psi}_{17}-1\right)-\left(\frac{3}{2}{\psi}_{16}-\frac{1}{2}{\psi}_{17}\right){\Theta }_{110}+\left(\frac{15}{8}{\Theta }_{110}-\frac{1}{8}{\psi}_{17}\right){\Theta }_{020}\right]$$

The minimum MSE of ${\widehat{\mathtt{F}}}_{SM}$ are given by:

28 $$\mathrm{MSE}\left({\widehat{\mathtt{F}}}_{SM}\right)\cong \frac{{\mathtt{F}}^{2}\left(y\right)}{16}\left[\frac{\begin{array}{c}64{\Theta }_{110}^{2}{\Theta }_{200}-48{\Theta }_{110}{\Theta }_{020}-128{\Theta }_{110}{\Theta }_{200}{\Theta }_{020}+48{\Theta }_{110}{\Theta }_{020}^{2}+64{{\Theta }_{200}\Theta }_{020}^{2}\\+64{\Theta }_{200}{\Theta }_{020}^{2}+9{\Theta }_{020}^{3} +64{\Theta }_{110}^{2}-64{\Theta }_{200}{\Theta }_{020}\end{array}}{16{\Theta }_{110}^{2}-16{\Theta }_{110}{\Theta }_{020}-4{\Theta }_{200}{\Theta }_{020}+{\Theta }_{020}^{2}-4{\Theta }_{020}}\right].$$

## Proposed estimator

The performance of the estimator can be is increased by utilizing the proper use of the auxiliary variable at estimation or designing stages or both stages. Ahmad et al.^24^ proposed an improved estimator based on cumulative distribution function (DF) under stratified random sampling using the dual auxiliary information. Taking motivation from Ahmad et al.^24^, we propose a novel class of estimators using DF of the study and auxiliary variables as well as mean of the usual auxiliary variable. The main benefit of our proposed generalised class of estimators is that it is more versatile and productive in terms of efficiency than the existing estimator. The proposed estimator is given by29 where  =  $\frac{1}{4}\left(\frac{\mathtt{F}\left(x\right)}{\widehat{\mathtt{F}}\left(x\right)}+\frac{\widehat{\mathtt{F}}\left(x\right)}{\mathtt{F}\left(x\right)}\right){\left(\mathrm{exp}\left(\frac{\mathtt{F}(x)-\widehat{\mathtt{F}}(x)}{\mathtt{F}(x)+\widehat{\mathtt{F}}(x)}\right)+\mathrm{exp}\left(\frac{\widehat{\mathtt{F}}(x-\mathtt{F}(x))}{\widehat{\mathtt{F}}(x+\mathtt{F}(x)}\right)\right)}^{\Upsilon}$ where ${\psi}_{18}$ and ${\psi}_{19}$ are the unknown constants, $\Upsilon$ is a suitably chosen constant.

After simplification of ${\widehat{\mathtt{F}}}_{Prop}$ , we have

30 $$\begin{aligned}{\widehat{\mathtt{F}}}_{Prop} & = \left[{\psi}_{18}\mathtt{F}\left(y\right)(1+{\xi }_{0})\left\{\frac{1}{4}\left\{{\left(1+{\xi }_{1}\right)}^{-1}+1+{\xi }_{1}\right\}{\left(\mathrm{exp}\left\{-\frac{1}{2} {\xi }_{1}{\left(1+\frac{1}{2}{\xi }_{1}\right)}^{-1}\right\} \right. }\right.\right. \\&\quad \left.\left. {\left. + \mathrm{exp}\left\{\frac{1}{2} {\xi }_{1}{\left(1+\frac{1}{2}{\xi }_{1}\right)}^{-1}\right\}\right)}^{\Upsilon}-{\psi}_{19}\overline{X}{\xi }_{1}\right\}\right]\left\{\mathrm{exp}\left(-\frac{1}{2} {\xi }_{1}{\left(1+\frac{1}{2}{\xi }_{1}\right)}^{-1}\right)\right\}\end{aligned}$$

Expanding (30), we get

$${\widehat{\mathtt{F}}}_{Prop}=\left[{\psi}_{18}\mathtt{F}\left(y\right)\left(1+{\xi }_{0}\right){\left\{\frac{1}{4}\left(\left(2+{\xi }_{1}^{2}\right)\left(2+\frac{1}{4}{\xi }_{1}^{2}\right)\right)\right\}}^{\Upsilon}-{\psi}_{19}\overline{X}{\xi }_{1}\right]\mathrm{exp}\left(1-\frac{1}{2} {\xi }_{1}+\frac{3}{8} {\xi }_{1}^{2}\right)$$

31 $${\widehat{\mathtt{F}}}_{Prop}-\mathtt{ }\mathtt{F}\left(y\right)=-\mathtt{ }\mathtt{F}\left(y\right)+{\psi}_{18}\mathtt{F}\left(y\right)\left[1+{\xi }_{0}-\frac{1}{2}{R\xi }_{1}-\frac{1}{2}{\xi }_{0}{\xi }_{1}+\Phi {\xi }_{1}^{2}+{\psi}_{19}R{\xi }_{1}^{2}\right],$$

where $$\Phi$$ = $$\frac{5}{8}$$ $$\Upsilon$$+$$\frac{3}{8}$$ , $$R$$ = $$\frac{{S}_{\mathtt{F}(y) }^{2}}{{\overline{X} }^{2}}$$

By taking expectations of (31) we get the bias of ${\widehat{\mathtt{F}}}_{Prop}$:

32 $${\text{Bias}}(\hat{\user2{F}}_{Prop} ) = - {\varvec{F}}\left( y \right) + \psi_{18} {\varvec{F}}\left( y \right)\left[ {1 - \frac{1}{2}\Theta_{110} + \Phi \Theta_{020} + \psi_{19} R\Theta_{020} } \right]$$

Taking expectations of (31), we obtain the required MSE:

33 $$\begin{aligned} {\text{MSE}}(\hat{\user2{F}}_{Prop} ) & = \psi_{18}^{2} \psi_{19}^{2} {\varvec{F}}^{2} \left( y \right){\varvec{F}}^{2} \left( x \right)\lambda C_{{x{ }}}^{2} - 2\psi_{18} {\varvec{F}}^{2} \left( y \right)\lambda C_{{x{ }}}^{2} + \frac{9}{4}\psi_{18}^{2} {\varvec{F}}^{2} \left( y \right){\uplambda } C_{{x{ }}}^{2} \\ & \quad + \psi_{18}^{2} {\varvec{F}}^{2} \left( y \right)\lambda C_{{{\varvec{F}}\left( y \right){ }}}^{2} + \psi_{18} {\varvec{F}}^{2} \left( y \right)\lambda \rho_{{{\varvec{F}}\left( y \right){\varvec{F}}\left( x \right)}} C_{{{\varvec{F}}\left( y \right)}} C_{{{\varvec{F}}\left( x \right)}}\\ & \quad - {2}\psi_{18}^{2} \rho_{{{\varvec{F}}\left( y \right){\varvec{F}}\left( x \right)}} C_{{{\varvec{F}}\left( y \right)}} C_{{{\varvec{F}}\left( x \right)}} + \psi_{18} \psi_{19} \overline{X}\lambda C_{{{\varvec{F}}\left( y \right)}} C_{x} \rho_{{{\varvec{F}}\left( y \right)x}} \\ & \quad - \psi_{18}^{2} {\varvec{F}}^{2} \left( y \right){ }\psi_{19} \overline{X}\lambda C_{{{\varvec{F}}\left( y \right)}} C_{x} \rho_{{{\varvec{F}}\left( y \right)x}} - {\varvec{F}}^{2} \left( y \right) - 2\psi_{18} {\varvec{F}}^{2} \left( y \right) + \psi_{18}^{2} {\varvec{F}}^{2} \left( y \right) \\ \end{aligned}$$

The optimal values of ${\psi}_{18}$ and ${\psi}_{19}$ are obtained from (33), is given by:

$${\psi}_{18}=\left[\frac{2\left\{{\uplambda C}_{\mathtt{F}\left(y\right)}^{2}{\rho }_{\mathtt{F}\left(y\right)x}^{2} +{\rho }_{\mathtt{F}(y)\mathtt{F}(x)}{C}_{\mathtt{F}(y)}{C}_{\mathtt{F}(x)}-2\uplambda {C}_{x }^{2}\right\}}{4{\uplambda C}_{\mathtt{F}\left(y\right)}^{2}{\rho }_{\mathtt{F}\left(y\right)x}^{2} +8{\uplambda \rho }_{\mathtt{F}(y)\mathtt{F}(x)}{C}_{\mathtt{F}(y)}{C}_{\mathtt{F}(x)}-4{\uplambda C}_{\mathtt{F}\left(y\right)}^{2}-9{C}_{x }^{2}-4}\right],$$

and

$${\psi}_{19}=-\frac{1}{4}\left[\frac{\left\{{4\uplambda \rho }_{\mathtt{F}(y)\mathtt{F}(x)}{C}_{\mathtt{F}(y)}{C}_{\mathtt{F}(x)} -4{\uplambda C}_{\mathtt{F}\left(y\right)}^{2}-\uplambda {C}_{\overline{X} }^{2}\right\}{C}_{\mathtt{F}(y)}{\rho }_{\mathtt{F}(y)x}}{\left({\uplambda C}_{\mathtt{F}\left(y\right)}^{2}{\rho }_{\mathtt{F}\left(y\right)x}^{2} +{\uplambda \rho }_{\mathtt{F}(y)\mathtt{F}(x)}{C}_{\mathtt{F}(y)}{C}_{\mathtt{F}(x)}-2{\uplambda C}_{x }^{2}-2\right)\overline{X}{C }_{x}}\right]$$

Putting the optimal values of ${\psi}_{18}$ and ${\psi}_{19}$ in (33) we get the minimum MSE as given by:

34 $${\mathrm{MSE}({\widehat{\mathtt{F}}}_{Prop}) }_{min}\cong {\mathtt{F}}^{2}(y)\left[1-\frac{{\Theta }_{020}}{4}-\frac{{\left\{1+\Phi {\Theta }_{020}-\frac{1}{2}{\Theta }_{020}\right\}}^{2}}{1+\left(2\Phi -\frac{3}{4}\right){\Theta }_{020}+{\Theta }_{200}-\frac{{\Theta }_{110}^{2}}{{\Theta }_{020}}}\right]$$

### Some special cases of our proposed estimator

*Case 1*: When $\Upsilon$ = 1 in (29), we have:

35 $$\begin{aligned}{\widehat{\mathtt{F}}}_{Prop1}& = \left[{\psi}_{18}{\widehat{\mathtt{F}}}_{(y)}\left\{\frac{1}{4}\left(\frac{\mathtt{F}\left(x\right)}{\widehat{\mathtt{F}}\left(x\right)}+\frac{\widehat{\mathtt{F}}\left(x\right)}{\mathtt{F}\left(x\right)}\right)\left(\mathrm{exp}\left(\frac{\mathtt{F}(x)-\widehat{\mathtt{F}}(x)}{\mathtt{F}(x)+\widehat{\mathtt{F}}(x)}\right)\right. \right. \right.\\&\quad \left. \left. \left. + \mathrm{exp}\left(\frac{\widehat{\mathtt{F}}(x-\mathtt{F}(x))}{\widehat{\mathtt{F}}(x+\mathtt{F}(x)}\right)\right)+{\psi}_{19}\left(\overline{X }-\overline{x }\right)\right\}\right]\mathrm{ exp}\left(\frac{\mathtt{F}(x)-\widehat{\mathtt{F}}(x)}{\mathtt{F}(x)+\widehat{\mathtt{F}}(x)}\right)\end{aligned}$$

The optimum values of ${\psi}_{18}$ and ${\psi}_{19}$ , are given by:

$${\psi}_{18}=\left[\frac{2\left\{{\uplambda C}_{\mathtt{F}\left(y\right)}^{2}{\rho }_{\mathtt{F}\left(y\right)x}^{2} +{\uplambda \rho }_{\mathtt{F}(y)\mathtt{F}(x)}{C}_{\mathtt{F}(y)}{C}_{\mathtt{F}(x)}-2\uplambda {C}_{x }^{2}-2\right\}}{4{\uplambda C}_{\mathtt{F}\left(y\right)}^{2}{\rho }_{\mathtt{F}\left(y\right)x}^{2} +8{\uplambda \rho }_{\mathtt{F}(y)\mathtt{F}(x)}{C}_{\mathtt{F}(y)}{C}_{\mathtt{F}(x)}-4{\uplambda C}_{\mathtt{F}\left(y\right)}^{2}-9{\uplambda C}_{x }^{2}-4}\right]$$

$${\psi}_{19}=-\frac{1}{4}\left[\frac{\left\{{4\uplambda \rho }_{\mathtt{F}(y)\mathtt{F}(x)}{C}_{\mathtt{F}(y)}{C}_{\mathtt{F}(x)} -4{\uplambda C}_{\mathtt{F}\left(y\right)}^{2}-\uplambda {C}_{x }^{2}+4\right\}{C}_{\mathtt{F}(y)}{\rho }_{\mathtt{F}(y)x}}{\left({\uplambda C}_{\mathtt{F}\left(y\right)}^{2}{\rho }_{\mathtt{F}\left(y\right)x}^{2} +{\uplambda \rho }_{\mathtt{F}(y)\mathtt{F}(x)}{C}_{\mathtt{F}(y)}{C}_{\mathtt{F}(x)}-2{\uplambda C}_{x }^{2}-2\right)\overline{X}{C }_{x}}\right]$$

Putting values of ${\psi}_{18}$ and ${\psi}_{19}$, we have:

36 $$\begin{aligned} & {\mathrm{MSE}({\widehat{\mathtt{F}}}_{Prop1}) }_{min}\cong {\mathtt{F}}^{2}(y)\\&\left[\frac{\uplambda {\mathtt{F}}^{2}\left(y\right)\left({\uplambda C}_{\mathtt{F}\left(y\right)}^{2}{\rho }_{\mathtt{F}\left(y\right)x}^{2}\left(\left({ C}_{\mathtt{F}\left(y\right)}^{2}{\rho }_{\mathtt{F}\left(y\right)\mathtt{F}\left(x\right)}^{2}-\frac{7}{4}{C}_{x }^{2}{\rho }_{\mathtt{F}\left(y\right)x}^{2}\right)\uplambda +{\rho }_{\mathtt{F}\left(y\right)x}^{2}-4\right)\right){ C}_{\mathtt{F}\left(y\right)}^{2}-4{ \rho }_{\mathtt{F}(y)\mathtt{F}(x)}{C}_{\mathtt{F}(y)}{C}_{\mathtt{F}\left(x\right)\left(\uplambda {C}_{x }^{2}-1\right){C}_{\mathtt{F}\left(y\right)}+4\uplambda {C}_{\mathtt{F}(x) }^{2}-{C}_{x }^{2}}}{4\left(\left(4{\rho }_{\mathtt{F}\left(y\right)x}^{2}-4\right){\uplambda C}_{\mathtt{F}\left(y\right)}^{2}+8{\uplambda \rho }_{\mathtt{F}(y)\mathtt{F}(x)}{C}_{\mathtt{F}(y)}{C}_{\mathtt{F}(x)}-9{\uplambda C}_{x }^{2}-4\right)}\right]\end{aligned}$$

*Case 2*: When $\Upsilon$ = 2 in (29), we have:

37 $$\begin{aligned}{\widehat{\mathtt{F}}}_{Prop2} & = \left[{\psi}_{18}{\widehat{\mathtt{F}}}_{(y)}\left\{\frac{1}{4}\left(\frac{\mathtt{F}\left(x\right)}{\widehat{\mathtt{F}}\left(x\right)}+\frac{\widehat{\mathtt{F}}\left(x\right)}{\mathtt{F}\left(x\right)}\right){\left(\mathrm{exp}\left(\frac{\mathtt{F}(x)-\widehat{\mathtt{F}}(x)}{\mathtt{F}(x)+\widehat{\mathtt{F}}(x)}\right)\right.}\right.\right.\\&\quad\left.\left.{\left.+\mathrm{exp}\left(\frac{\widehat{\mathtt{F}}(x-\mathtt{F}(x))}{\widehat{\mathtt{F}}(x+\mathtt{F}(x)}\right)\right)}^{2}+{\psi}_{19}\left(\overline{X }-\overline{x }\right)\right\}\right]\mathrm{ exp}\left(\frac{\mathtt{F}(x)-\widehat{\mathtt{F}}(x)}{\mathtt{F}(x)+\widehat{\mathtt{F}}(x)}\right)\end{aligned}$$

The values of ${\psi}_{18}$ and ${\psi}_{19}$ , are given by:

$${\psi}_{18}=\frac{1}{4}\left[\frac{\left\{{-4\uplambda C}_{\mathtt{F}\left(y\right)}^{2}{\rho }_{\mathtt{F}\left(y\right)x}^{2}-4\uplambda {\rho }_{\mathtt{F}\left(y\right)\mathtt{F}\left(x\right)}{C}_{\mathtt{F}\left(y\right)}{C}_{\mathtt{F}\left(x\right)}+13{\uplambda C}_{\mathtt{F}\left(x\right)}^{2}+8\right\}}{-2{\uplambda C}_{\mathtt{F}\left(y\right)}^{2} {\rho }_{\mathtt{F}\left(y\right)x}^{2} -4{\uplambda \rho }_{\mathtt{F}\left(y\right)\mathtt{F}\left(x\right)}{C}_{\mathtt{F}\left(y\right)}{C}_{\mathtt{F}\left(x\right)}+7{\uplambda C}_{\mathtt{F}\left(x\right)}^{2}+2{\uplambda C}_{\mathtt{F}\left(y\right)}^{2}+2{\uplambda C}_{\mathtt{F}\left(y\right)}^{2}+2}\right]$$

$${\psi}_{19}=-\left[\frac{\left\{{-4\uplambda \rho }_{\mathtt{F}\left(y\right)\mathtt{F}\left(x\right)}{C}_{\mathtt{F}\left(y\right)}{C}_{\mathtt{F}\left(x\right)}+{\uplambda C}_{\mathtt{F}\left(x\right)}^{2}+4{\uplambda C}_{\mathtt{F}\left(y\right)}^{2}-4\right\}{C}_{\mathtt{F}(y)}{\rho }_{\mathtt{F}(y)x}}{\left(-4{\uplambda C}_{\mathtt{F}\left(y\right)}^{2} {\rho }_{\mathtt{F}\left(y\right)x}^{2} -4{\uplambda \rho }_{\mathtt{F}\left(y\right)\mathtt{F}\left(x\right)}{C}_{\mathtt{F}\left(y\right)}{C}_{\mathtt{F}\left(x\right)}+13{\uplambda C}_{\mathtt{F}\left(x\right)}^{2}+8\right)\overline{X}{C }_{x}}\right]$$

Putting values of ${\psi}_{18}$ and ${\psi}_{19}$, we have:38 where  = $\frac{16}{169}{\uplambda C}_{\mathtt{F}\left(y\right)}^{2}$(${\rho }_{\mathtt{F}\left(y\right)\mathtt{F}\left(x\right)}^{2}$ $-$ 3 ${\rho }_{\mathtt{F}\left(y\right)\overline{X} }^{2}$) 

 = $$-$$ $$\frac{8}{13}$$($${\uplambda C}_{\mathtt{F}\left(x\right)}^{2}-\frac{8}{13}$$)$${ \rho }_{\mathtt{F}\left(y\right)\mathtt{F}\left(x\right)}{C}_{\mathtt{F}\left(y\right)}{C}_{\mathtt{F}\left(x\right)}$$

 = 128 $${\uplambda \rho }_{\mathtt{F}\left(y\right)\mathtt{F}\left(x\right)}{C}_{\mathtt{F}\left(y\right)}{C}_{\mathtt{F}\left(x\right)}$$

## Numerical study

We carry out a numerical analysis utilizing three real data sets to found the efficiency of our proposed estimator. In terms of percentage relative efficiency, we assess how well our proposed estimator performs in comparison to its existing estimators. The following expression was used to calculate the percentage relative efficiency (PRE).

$$\mathrm{PRE}=\frac{{Var(\widehat{\mathtt{F}}}_{usual})}{MSE({\widehat{\mathtt{F}}}_{i})}\times 100$$

where (*i* = *R, P*,…,Prop1,Prop2).

Population-I: [Source: Punjab Bureau of Statistics (2021–2022)].

*Y* = Covid-19 test performed in Punjab district during 2021.

*X* = Covid-19 confirmed cases in Punjab districts during 2021.

*N* = 228, *n* = 40.

Population-II: [Source: Source: Punjab Bureau of statistics (2021–2022)].

*Y* = Total number of beds in 30th June 2021.

*X* = Total allocated beds for Covid.

*N* = 36, *n* = 8.

Population-III: [Source: Herbert (2009)].

http://archibe.ics.uci.edu/ml/datasets/wine

*Y* = Aspartame.

*X* = Leucine.

## Simulation study

We have generated population of size 5000 from a bivariate normal distribution with different covariance matrices. The population means and covariance matrices, are given below:

### Population-I

$$\mu =\left[\begin{array}{l}2\\ 2\end{array}\right]$$

and

$$\sum { = \left[ {\begin{array}{*{20}l} 3 & 3 \\ 3 & 6 \\ \end{array} } \right]}$$

$${\rho }_{\mathtt{F}(y)\mathtt{F}(x)}$$ = 0.5704609, $${\rho }_{\mathtt{F}(x)x}$$ = 0.56466576 and $${\rho }_{\mathtt{F}(y)x}$$ = 0.8000432

### Population-II

$$\mu =\left[\begin{array}{l}2\\ 2\end{array}\right]$$

and

$$\sum { = \left[ {\begin{array}{*{20}l} 3 & {3.5} \\ {3.5} & 8 \\ \end{array} } \right]}$$

$${\rho }_{\mathtt{F}(y)\mathtt{F}(x)}$$ = 0.5687875, $${\rho }_{\mathtt{F}(x)x}$$ = 0.5015351 and $${\rho }_{\mathtt{F}(y)x}$$ = 0.7986237.

### Population-III

$$\mu =\left[\begin{array}{c}2\\ 2\end{array}\right]$$

and

$$\sum { = \left[ {\begin{array}{*{20}c} 3 & 3 \\ 3 & 5 \\ \end{array} } \right]}$$

$${\rho }_{\mathtt{F}(y)\mathtt{F}(x)}$$ = 0.7955929, $${\rho }_{\mathtt{F}(x)x}$$ = 0.7623945 and $${\rho }_{\mathtt{F}(y)x}$$ = 0.8042652.

## Discussion

We used three real populations and simulation to assess the efficiency of our proposed novel generalized class of estimators. We also considered different sample size from the populations. In our study we used a variety of data sets where we have a good mix of correlations between the study variable and the auxiliary variable. The data descriptions of real data sets are given in Table 1. The numerical results of MSE and PRE established on real data sets are presented in Tables 2 and 3. Additionally, it is emphasized that based on the numerical illustration the proposed estimators are more efficient than the existing estimators. It is observed that the proposed estimator is appreciable in terms of smallest MSE and greater PRE as compared to existing counterparts. The mean square error and PRE results based on simulated data sets are given in Tables 4 and 5. Thus it is recommended that the proposed estimators are useful in practice.

**Table 1:** Summary statistics using real data sets.

Parameters	Population-I	Population-II	Population-III
*N*	228	36	67
*n*	40	8	12
$\Lambda$	0.02061	0.09722	0.06840796
$\mathtt{F}\left(y\right)$	0.50000	0.50000	0.4925373
$\mathtt{F}\left(x\right)$	0.50000	0.50000	0.4925373
$\overline{X }$	882.93420	215.6389	20.59851
${\rho }_{\mathtt{F}\left(y\right)\mathtt{F}\left(x\right)}$	0.5789474	0.3333333	0.6417112
${\rho }_{\mathtt{F}\left(y\right)x}$	0.2369336	0.3830227	0.6654633
${\rho }_{\mathtt{F}\left(x\right)x}$	0.2552666	0.4224452	0.7896493
${C}_{\mathtt{F}\left(y\right)}$	1.002200	1.0141850	1.022699
${C}_{\mathtt{F}\left(x\right)}$	1.002200	1.0141850	1.0226990
${C}_{x}$	3.482949	1.5045280	0.6279163

**Table 2:** Mean square error using real data sets.

Estimators	Population-I	Population-II	Population-III
${\widehat{\mathtt{F}}}_{usual}$	0.005176211	0.02500000	0.01735724
${\widehat{\mathtt{F}}}_{R}$	0.01634593	0.03333333	0.01243700
${\widehat{\mathtt{F}}}_{P}$	0.01634593	0.06666667	0.05699116
${\widehat{\mathtt{F}}}_{HS,1}$	0.00345985	0.023312331	0.01035522
${\widehat{\mathtt{F}}}_{HS,2}$	0.003442017	0.02226877	0.01024069
${\widehat{\mathtt{F}}}_{Reg}$	0.003441249	0.02222220	0.01020965
${\widehat{\mathtt{F}}}_{BTR}$	0.00347351	0.02291667	0.01055822
${\widehat{\mathtt{F}}}_{BTP}$	0.009467018	0.03958333	0.03283489
${\widehat{\mathtt{F}}}_{SG}$	0.003370345	0.01975446	0.009547592
${\widehat{\mathtt{F}}}_{SW}$	0.003441249	0.02222220	0.01020965
${\widehat{\mathtt{F}}}_{C}$	0.003441249	0.02222220	0.01020965
${\widehat{\mathtt{F}}}_{YG}$	0.003393549	0.02022472	0.009766925
${\widehat{\mathtt{F}}}_{YS}$	0.003354136	0.01936910	0.009353307
${\widehat{\mathtt{F}}}_{SM}$	0.003124916	0.01585976	0.006853708
${\widehat{\mathtt{F}}}_{Prop1(\Upsilon=1 )}$	0.001843958	0.01282890	0.005831504
${\widehat{\mathtt{F}}}_{Prop2(\Upsilon=2 )}$	0.0006506061	0.00157237	0.00558592

**Table 3:** PRE using real data sets.

Estimators	Population-I	Population-II	Population-III
${\widehat{\mathtt{F}}}_{usual}$	100	100	100
${\widehat{\mathtt{F}}}_{R}$	118.75	75.0000	139.5522
${\widehat{\mathtt{F}}}_{P}$	31.66667	37.5000	30.45603
${\widehat{\mathtt{F}}}_{HS,1}$	149.608	107.2395	167.6183
${\widehat{\mathtt{F}}}_{HS,2}$	150.3831	112.2649	169.4930
${\widehat{\mathtt{F}}}_{Reg}$	150.4167	112.5000	170.0083
${\widehat{\mathtt{F}}}_{BTR}$	149.0196	109.0909	164.3956
${\widehat{\mathtt{F}}}_{BTP}$	54.67626	63.15789	52.86219
${\widehat{\mathtt{F}}}_{SG}$	153.5811	126.5537	181.7971
${\widehat{\mathtt{F}}}_{SW}$	150.4167	112.5000	170.0083
${\widehat{\mathtt{F}}}_{C}$	150.4167	112.5000	170.0083
${\widehat{\mathtt{F}}}_{YG}$	152.5309	123.6111	177.7145
${\widehat{\mathtt{F}}}_{YS}$	154.3232	129.0715	185.5733
${\widehat{\mathtt{F}}}_{SM}$	165.6432	157.6317	253.2533
${\widehat{\mathtt{F}}}_{Prop1(\Upsilon=1 )}$	280.712	194.8573	297.6461
${\widehat{\mathtt{F}}}_{Prop2(\Upsilon=2 )}$	795.5984	1589.956	312.2597

**Table 4:** MSE using simulated data sets.

Estimators	Population-I	Population-II	Population-III
${\widehat{\mathtt{F}}}_{usual}$	0.006201073	0.006199319	0.0003666761
${\widehat{\mathtt{F}}}_{R}$	0.006429300	0.004080697	0.0004166983
${\widehat{\mathtt{F}}}_{P}$	0.018840000	0.020826200	0.0010823830
${\widehat{\mathtt{F}}}_{HS,1}$	0.004744011	0.003403407	0.0002944911
${\widehat{\mathtt{F}}}_{HS,2}$	0.004704825	0.003323658	0.0002943374
${\widehat{\mathtt{F}}}_{Reg}$	0.004704767	0.003397053	0.0002943372
${\widehat{\mathtt{F}}}_{BTR}$	0.004706791	0.003576476	0.0002959711
${\widehat{\mathtt{F}}}_{BTP}$	0.010912140	0.011949230	0.0006288133
${\widehat{\mathtt{F}}}_{SG}$	0.004577778	0.003351978	0.0002938594
${\widehat{\mathtt{F}}}_{SW}$	0.004704767	0.003397053	0.0002943372
${\widehat{\mathtt{F}}}_{C}$	0.004704767	0.003397053	0.0002943372
${\widehat{\mathtt{F}}}_{YG}$	0.00461467	0.003302443	0.0002939996
${\widehat{\mathtt{F}}}_{YS}$	0.004553616	0.002966457	0.0002937858
${\widehat{\mathtt{F}}}_{SM}$	0.004233409	0.003339358	0.0002926822
${\widehat{\mathtt{F}}}_{Prop1(\Upsilon=1 )}$	0.003646903	0.002966456	0.0002492984
${\widehat{\mathtt{F}}}_{Prop2(\Upsilon=2 )}$	0.000665874	0.001949947	3.170472e-06

**Table 5:** PRE using simulated data sets.

Estimators	Population-I	Population-II	Population-III
${\widehat{\mathtt{F}}}_{usual}$	100	100	100
${\widehat{\mathtt{F}}}_{R}$	96.4502	151.9181	87.99558
${\widehat{\mathtt{F}}}_{P}$	32.91439	29.76692	33.87675
${\widehat{\mathtt{F}}}_{HS,1}$	130.7137	181.7912	124.5118
${\widehat{\mathtt{F}}}_{HS,2}$	131.8024	182.1504	124.5768
${\widehat{\mathtt{F}}}_{Reg}$	131.8004	182.4911	124.5769
${\widehat{\mathtt{F}}}_{BTR}$	131.7473	173.3359	123.8892
${\widehat{\mathtt{F}}}_{BTP}$	56.82727	51.88051	58.3124
${\widehat{\mathtt{F}}}_{SG}$	135.4603	186.521	124.7794
${\widehat{\mathtt{F}}}_{SW}$	131.804	182.4911	124.5769
${\widehat{\mathtt{F}}}_{C}$	131.8024	182.4911	124.5769
${\widehat{\mathtt{F}}}_{YG}$	134.3774	184.9451	124.72
${\widehat{\mathtt{F}}}_{YS}$	136.1791	187.719	124.8107
${\widehat{\mathtt{F}}}_{SM}$	146.4794	185.644	125.2813
${\widehat{\mathtt{F}}}_{Prop1(\Upsilon=1 )}$	170.0367	208.9806	147.0832
${\widehat{\mathtt{F}}}_{Prop2(\Upsilon=2 )}$	902.6828	317.9225	11,565.35

Moreover, we have also checked that the proposed generalized class of estimator is consistent when estimating the finite population DF; that is *n* increases; the values of proposed estimators get closer and closer to the true value. The researchers are thus recommended that the proposed estimators may be preferred over the existing estimators for the use of practical applications.

## Conclusion

In this article, we suggested a new class of estimators for estimation of the finite population DF based on simple random sampling, which utilizes DF of the study and the auxiliary variables as well as also mean of the auxiliary variable. Numerical expressions of the bias and mean squared errors are derived up to first order of approximation. We used three real data sets, and have been determined from numerical results, that the proposed estimators perform well as compared to existing estimators. The results of the simulation study also confirmed the efficiency of the proposed estimators. We generated two new estimators from our suggested class of estimators. The gain in efficiency of ${\widehat{\mathtt{F}}}_{Prop2}$ is more as compared to ${\widehat{\mathtt{F}}}_{Prop1}$. The current work can be extended to generate a better family of estimators for simple and stratified random sampling for estimating the DF based on measurement errors, two-stage and three-stage sampling designs.

## Data Availability

All data generated or analysed during this study are included in this published article.
